# Urinary exosomes aggravate diabetic kidney disease by inducing podocyte ferroptosis via the miR‐217/SIRT1/Nrf2 pathway

**DOI:** 10.1002/ccs3.70076

**Published:** 2026-04-25

**Authors:** Xin Du, Yubo Jiang, Hongbin Guo, Haiying Zhang, Shaoqing Wang, Hao Deng, Xinyi Huang, Xiu Li

**Affiliations:** ^1^ Department of Endocrine NanBu County People's Hospital Nanchong Sichuan Province China; ^2^ Department of Nephrology The Second Affiliated Hospital of Chengdu Medical College Nuclear Industry 416 Hospital Chengdu Sichuan Province China; ^3^ Department of Clinical Medicine School of Clinical Medicine Chengdu Medical College Chengdu Sichuan Province China; ^4^ Department of Endocrine Metabolism The 3RD Affiliated Hospital of Chengdu Medical College Chengdu Pidu District People's Hospital Chengdu Sichuan Province China; ^5^ Department of Nephrology The First Affiliated Hospital of Chengdu Medical College Chengdu Sichuan Province China; ^6^ Development and Regeneration Key Laboratory of Sichuan Province School of Basic Medical Sciences Chengdu Medical College Chengdu Sichuan Province China

**Keywords:** diabetic kidney disease, ferroptosis, miR‐217, SIRT1/Nrf2 pathway, urinary exosomes

## Abstract

Urinary exosomal microRNAs (miRNAs) mediate intercellular communication in diabetic kidney disease (DKD), a leading contributor to end‐stage renal failure. However, the involvement of urinary exosomal miR‐217 in DKD remains poorly understood. Urinary exosomes were characterized, and miR‐217 expression was measured in clinical samples. The miR‐217/SIRT1 interaction was validated by dual‐luciferase assays. Podocyte viability, ferroptosis‐related markers, and protein expression were assessed in vitro, whereas renal function and histology were evaluated in a streptozotocin‐induced DKD mouse model. MiR‐217 was upregulated in urinary exosomes derived from patients with DKD. Inhibition of miR‐217 alleviated exosome‐induced podocyte injury, lipid peroxidation, and ferroptosis, and preserved podocyte markers; these protective effects can be partially reversed by Fer‐1 or miR‐217 inhibition. SIRT1 was confirmed to be a direct target of miR‐217, which negatively regulated SIRT1 expression and suppressed the SIRT1/Nrf2 pathway. SIRT1 knockdown abolished the protective effects of miR‐217 inhibition. Conversely, miR‐217 mimic exacerbated ferroptotic damage and downregulated the expression of podocyte markers, which were partly rescued by SIRT1 overexpression. In vivo, miR‐217 inhibition attenuated DKD‐exosome‐aggravated kidney injury and ferroptosis, whereas SIRT1 inhibition abrogated this protective effect. Collectively, these findings indicate that urinary exosomal miR‐217 promotes podocyte ferroptosis and DKD progression via suppression of the SIRT1/Nrf2 pathway, suggesting a potential therapeutic target for DKD.

## Graphical abstract

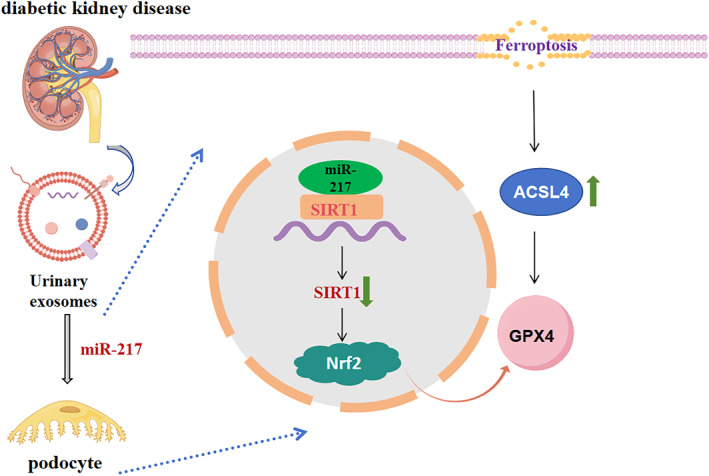

## 1 INTRODUCTION

Diabetic kidney disease (DKD) is the primary contributor to end‐stage renal disease (ESRD) globally, affecting approximately 30%–40% of patients with type 2 diabetes mellitus.^1^ The predominant clinical features of DKD involve persistent albuminuria, glomerulosclerosis, and a decreased glomerular filtration rate.^2^ At present, it is generally believed that persistent high glucose environment, oxidative stress, and inflammatory responses induce podocyte injury, thereby inducing proteinuria, which is the key cause of the evolution of DKD.^3^ As key elements of the glomerular filter, podocytes are highly differentiated epithelial cells that line the glomerular basement membrane and maintain its selective permeability.^4^ Although current therapies for DKD, such as weight loss, glycemic control, and blood pressure reduction, have shown limited efficacy in delaying the onset or progression of renal damage, the pathological mechanisms of DKD remains incompletely understood.^5^ Therefore, understanding the mechanisms driving podocyte injury and discovering new molecular pathways involved in the progression of DKD is crucial for developing effective therapeutic strategies.

Exosomes are small extracellular vesicles with a diameter of 30–150 nm that are secreted by various cell types, including renal cells.^5^ Accumulating evidence has indicated that exosomes are of great significance in intercellular communication and various biological processes by shuttling nucleic acids and proteins between cells.^6^ Urinary exosomes are particularly valuable for DKD research due to their noninvasive accessibility and their capacity to reflect the physiological state of renal tissue.^7^ Under high glucose conditions, the expression of various urinary exosomal miRNAs is significantly altered, and these miRNAs acts as key regulators of renal cell function. For example, Han et al. demonstrated that urinary exosomal miR‐145‐5p promotes podocyte apoptosis via targeting SRGAP2 and modulating the RhoA/ROCK pathway in DKD.^8^ Additionally, our previous research indicated that urinary exosomal miR‐516b‐5p exacerbates DKD by promoting inflammatory responses and activating the NLRP3 inflammasome via the SIRT3/AMPK pathway.^9^ Sun et al. suggested that miR‐217 expression is elevated in podocytes under high glucose stimulation; inhibition of miR‐217 reduced reactive oxygen species (ROS) levels and attenuated podocyte apoptosis, thereby mitigating high glucose‐induced cellular damage.^10^ However, the specific mechanism of urinary exosomal miR‐217 in DKD is still not fully understood.

Ferroptosis is an iron‐dependent form of programmed cell death characterized by the accumulation of intracellular ROS.^11^ Accumulating studies have shown that ferroptosis is closely related to the pathophysiological processes of a variety of diseases, including kidney injury.^12^ The levels of inflammatory factors and ROS are significantly elevated in the hyperglycemia state, leading to podocyte ferroptosis and damage to the barrier damage.^13^ In addition, specific inhibitors such as ferrostatin‐1 (Fer‐1) can effectively rescue ferroptosis, alleviate kidney injury, and reduce proteinuria, but the significance of ferroptosis in DKD remains to be elucidated.^14^ Sirtuin 1 (SIRT1) is a member of the NAD^+^‐dependent deacetylase Sirtuin family that is involved in regulating cell apoptosis, oxidative stress, and other processes. Zhou et al. demonstrated a functional association between SIRT1 and ferroptosis and found that SIRT1 inhibitor EX527 could attenuate the effect of naringenin on high glucose‐stimulated ferroptosis in HK‐2 cells.^15^ Besides, SIRT1 activates nuclear factor erythroid 2‐related factor 2 (Nrf2) and drives the antioxidant defense system to inhibit ferroptosis by upregulating glutathione peroxidase 4 (GPX4). Qiong et al. suggested that Irisin postconditioning attenuated acute kidney injury by inhibiting ferroptosis via SIRT1/Nrf2 pathway.^16^ However, the function of the SIRT1/Nrf2 pathway and ferroptosis in DKD pathogenesis is not fully understood.

Consequently, this research was designed to illustrate the expression of urinary exosomal miR‐217 in patients with DKD and to explore whether it is involved in the procession of DKD by regulating podocyte ferroptosis via the SIRT1/Nrf2 pathway. Our findings have the potential to offer a novel and promising therapeutic approach for DKD.

## 2 MATERIALS AND METHODS

### 2.1 Urinary exosome isolation and identification

Urine specimens were gathered from 15 subjects with diabetes mellitus (DM) patients, DKD patients, and healthy controls. Cellular debris was separated from the samples by centrifugation (3000 × g, 20 min, 4°C). Bacteria, residual cells, and residual debris were further eliminated by filtration through 0.22 μm filters. The resulting supernatant was concentrated to 10 mL using a centrifugal filter unit (Millipore, USA). Urinary exosomes from urine were isolated following the manufacturer's instructions with the exoEasy Maxi Kit (Qiagen, #76064). Total exosomal protein was quantified via BCA Protein Assay Kit (beyotime, P0012). The morphology, size distribution, and concentration of isolated exosomes were characterized by transmission electron microscopy (TEM) and nanoparticle tracking analysis (NTA). The expression of exosomal markers (CD9 and CD63) and the endoplasmic reticulum protein Calnexin was analyzed by Western blot. For exosome internalization assays, exosomes were labeled with the fluorescent dye PKH26 via the PKH26 Red Fluorescent Cell Linker Mini Kit (Sigma‐Aldrich, MINI26). MPC5 cells were incubated with a culture medium containing PKH26‐labeled exosomes for 12 h. Thereafter, the cells were fixed, and nuclei were stained with DAPI (Solarbio, #D8200). Internalization of exosomes was visualized using confocal fluorescence microscopy.

### 2.2 Cell culture

The mouse podocyte cell line MPC5 was provided from Procell (CL‐0855, Wuhan, RRID: CVCL_AS87) and cultivated in DMEM (PM150210, Procell) containing 10% FBS (Gibco) and 1% penicillin‐streptomycin (PB180120, Procell) at 37°C in a humidified atmosphere with 5% CO_2_. The cell line was authenticated by short tandem repeat (STR) profiling prior to the described experiments. Besides, cell line was regularly tested and confirmed to be free of mycoplasma contamination using a PCR‐based method throughout the study.

### 2.3 Cell counting kit‐8 (CCK‐8) assay

MPC5 cells were inoculated into 96‐well plates at a density of 1 × 10^4^ cells/well. Following treatment, the cell viability of the podocytes was assessed via the CCK‐8 kit (P‐CA‐001, Sharebio). CCK‐8 solution (10 μL/well) was added and incubated for 2 h at 37°C, followed by absorbance measurement at 450 nm on a microplate reader. (Thermo, Multiskan MK3). The assay was independently repeated three times.

### 2.4 Cell treatment and transfection

The exosomes isolated from urine samples of the normal, DM, and DKD groups were named normal‐Exo, DM‐Exo, and DKD‐Exo, respectively. MPC5 cells were then exposed to these urinary exosomes (45 μg/mL) and incubated for 24 h prior to subsequent analysis. MiR‐217 mimic, miR‐217 inhibitor, and their corresponding negative controls (NC), as well as pcDNA‐SIRT1/pcDNA3.1 plasmids and SIRT1‐targeting shRNA (sh‐SIRT1) or scrambled shRNA (sh‐NC), were synthesized by GenePharma (Shanghai, China). The miR‐217 mimic (5′‐UACUGCAUCAGGAACUGAUUGGA‐3′), mimic control (5′‐UUCUCCGAACGUGUCACGUTT‐3′), miR‐217 inhibitor (5′‐UCCAAUCAGUUCCUGAUGCAGUA‐3′), and inhibitor control (5′‐GCCUCCGGCUUCGCACCUCU‐3′) were synthesized from GenePharma. All transfections were carried out by Lipofectamine™ 3000 (Invitrogen) following the manufacturer's protocol.

### 2.5 Lipid peroxidation assay

Intracellular ROS levels were assessed using BODIPY™ 581/591 C11 (D3861; Thermo Fisher Scientific). Briefly, pre‐treated MPC5 cells were washed with PBS, harvested, and resuspended at a density of 1 × 10^6^ cells/mL, followed by incubation with 2 μM C11‐BODIPY at 37°C for 30 min. After washing with PBS, cells were fixed with 4% paraformaldehyde, counterstained with DAPI, and observed using a confocal laser scanning microscope (Zeiss, Germany).

### 2.6 Measurement of MDA and GSH levels

MPC5 cells were cultured in 6‐well plates (1 × 10^5^ cells/well). After treatment, cells were collected and the supernatant was prepared based on the instructions. The Lipid Peroxidation MDA Assay Kit (S0131S, Solarbio) and intracellular glutathione (GSH) Detection Assay Kit (ab112132, Abcam) were used to analyze the levels of GSH and MDA.

### 2.7 Intracellular Fe^2+^ level

Intracellular iron (Fe^2+^) levels were measured using a commercial Iron Assay Kit (Abcam, ab83366) in accordance with the manufacturer's guidelines. In short, after treatment, cells were collected and washed three times with PBS. Then, 200 μL of Iron Assay Buffer was added to lyse the cells, and the lysate was collected. After 30 min of treatment with an iron reducer, an iron probe was introduced to the lysate for 60 min, followed by absorbance measurement at 593 nm (BioTek‐Epoch2).

### 2.8 Dual‐luciferase activity assay

The targeting relationship between miR‐217 and SIRT1 was verified by dual luciferase reporter assays. The wild‐type (WT) or mutant (MUT) 3′‐UTR of SIRT1, encompassing the miR‐217 binding site, was synthesized and inserted into the pmirGLO dual luciferase vector (Promega, USA). The WT sequence was 5′‐GCCAAGTGTTCATGTGCACTGA‐3′, and the MUT sequence was 5′‐GCCAAGTGTTCATGAGTCATGA‐3′, in which the seed region was mutated to disrupt miR‐217 binding. MPC5 cells were co‐transfected with inhibitor NC or miR‐217 inhibitor and constructed pmirGLO‐SIRT1‐WT or pmirGLO‐SIRT1‐MUT plasmids by Lipofectamine 3000 (Invitrogen). After 48 h, luciferase activity was detected based on the protocol of Dual‐Luciferase Reporter Assay System (E1910, Promega).

### 2.9 Real‐time qPCR analysis

Total RNA was isolated from exosomes or cells using total RNA extraction kit (R1200, Solarbio) and quantified on NanoDrop 2000 (Thermo Fisher Scientific, USA). TRUEscript 1st Strand cDNA Synthesis Kit (PC1802, Aidlab) was used for reverse transcription. qPCR was conducted on a CG real‐time PCR (Heal Force) using SYBR Premix Ex Taq Kit (TaKaRa). GAPDH was used as the internal control for SIRT1 mRNA, and U6 snRNA was used as the internal control for miR‐217. Primer sequences are provided in Table 1. Data were calculated using the 2^−ΔΔCt^ method.

**TABLE 1:** Primer sequences used in this study.

Gene	Forward	Reverse
miR‐217	5′‐CGCGTACTGCATCAGGAACTG‐3′	5′‐AGTGCAGGGTCCGAGGTATT‐3′
SIRT1	5′‐TGTTTCCTGTGGGATACCTGA‐3′	5′‐TGAAGAATGGTCTTGGGTCTTT‐3′
GAPDH	5′‐AGAAGGCTGGGGCTCATTTG‐3′	5′‐AGGGGCCATCCACAGTCTTC‐3′
U6	5′‐CTCGCTTCGGCAGCACA‐3′	5′‐AACGCTTCACGAATTTGCGT‐3′

### 2.10 Western blot analysis

RIPA Lysis Buffer (AR0102, BOSTER) was applied for total protein extraction. Protein concentration was assessed via BCA Protein Assay Kit (P0010S, Beyotime), and 40 μg of each sample was fractionated by 12% SDS‐PAGE and shifted to PVDF membranes (IPVH00010, Millipore). After blocking with 5% skim milk for 2 h, the membrane was incubated at 4°C with indicated antibodies, including anti‐CD9 (ab307085, Abcam, 1:1000), anti‐CD63 (#AF5117, Affinity, 1:1000), anti‐Calnexin (10427‐2‐AP, Proteintech, 1:5000), anti‐Nephrin (85845‐1‐RR, Proteintech, 1:1000), anti‐Podocin (#DF8593, Affinity, 1:1000), anti‐GPX4 (#DF6701, Affinity, 1:1000), anti‐ACSL4 (22401‐1‐AP, Proteintech, 1:5000), anti‐SIRT1 (ab189494, Abcam, 1:1000), anti‐Nrf2 (#AF0639, Affinity, 1:1000), and anti‐β‐actin (20536‐1‐AP, Proteintech, 1:5000). After that, the membranes were incubated with secondary antibodies (S0001, Affinity, 1:5000) and exposed to ECL reagent (P2300, ncmbio). All experiments were performed with three independent replicates.

### 2.11 Establishment of animal models and treatment

A total of 30 male C57BL/6 mice (6–8 weeks old, 20–22 g) were obtained from Chengdu Dossy Experimental Animal Co., Ltd. and maintained under SPF conditions with a 12‐h light/dark cycle, temperature of 23 ± 2°C, and humidity of 50 ± 5%. Food and water were provided freely. The mice were randomly assigned to five groups (*n* = 6/group): control, STZ, STZ + DKD‐Exo + inhibitor NC, STZ + DKD‐Exo + miR‐217 inhibitor, and STZ + DKD‐Exo + miR‐217 inhibitor + Ex‐527. After a 2‐day acclimatization period, animals in the STZ groups were fed a high‐fat diet. Three weeks later, DKD models were induced by intraperitoneal injection of streptozotocin (50 mg/kg; Sigma‐Aldrich) for five consecutive days. Control mice received a standard diet and an equivalent volume of citrate buffer. One week after the final streptozotocin injection, fasting blood glucose (FBG) levels >16.6 mmol/L were considered indicative of successful DKD modeling. Body weight was measured before the intervention and at the end of the 8‐week experimental period. Mice in the STZ + DKD‐Exo groups received daily tail vein injections of 100 μg DKD‐Exo (dissolved in 150 μL saline) and/or miR‐217 inhibitor (50 nmol/kg). Animals in the SIRT1 inhibitor group were administered Ex‐527 (10 mg/kg) via intraperitoneal injection. Control mice got the same amount of saline. After 8 weeks, all mice were euthanized under ether anesthesia, and kidney tissue was obtained for subsequent analysis.

### 2.12 Measurement of serum SCr and BUN levels

Blood specimens were gathered from euthanized mice, and serum was extracted by centrifugation at 12,000 rpm for 5 min. Renal function‐related parameters, including serum creatinine (Scr) and blood urea nitrogen (BUN), were determined following the instructions of the Creatinine (Cr) Assay Kit (C011‐2‐1, njjcbio) and the Urea Assay Kit (C013‐2‐1, njjcbio), respectively.

### 2.13 Hematoxylin‐eosin (H&E) staining

Renal tissues were fixed after excision in 4% formaldehyde. The kidney specimens underwent dehydration through a series of graded alcohols. Subsequently, they were embedded in paraffin and cut into 4 μm sections. Afterward, the sections were stained with hematoxylin for 5 min, rinsed with water, and subsequently counterstained with eosin for 3 min according to the protocol of Hematoxylin‐Eosin/HE Staining Kit (C02‐04004, Beyotime). Pathological changes were observed using a light microscope (Olympus, Japan).

### 2.14 Statistical analysis

Results were presented as means ± standard deviation (SD). Statistical analysis was conducted using GraphPad Prism 8.0 software (La Jolla, CA, USA). Comparisons between two groups were made using unpaired *t*‐tests. For comparisons among multiple groups, one‐way analysis of variance (ANOVA) with Tukey's post hoc test was used. *p* < 0.05 was deemed statistically significant.

## 3 RESULTS

### 3.1 Urinary exosome‐derived miR‐217 was highly expressed in DKD patients

**FIGURE 1:**
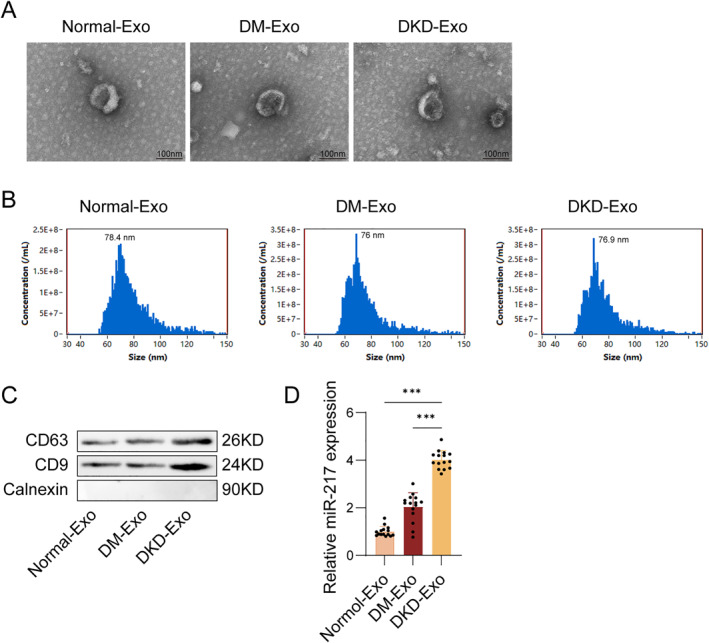
Characterization of urinary exosomes. (A) The morphology of exosomes was observed by transmission electron microscope (TEM). (B) Diameter sizes and their concentration distributions of exosomes were measured by nanoparticle tracking analysis (NTA). (C) Exosome markers (CD9, CD63, and Calnexin) were assessed using Western blot. (D) The mRNA level of miR‐217 in urinary exosomes was measured by qRT‐PCR analysis. *n* = 3, *p < 0.05, **p < 0.01.

Urinary exosomes were successfully isolated from clinical samples of DKD patients and characterized them using several methods. TEM showed a bilayer membrane structure with typical exosome morphology (Figure 1A). NTA revealed particle diameters of 130–150 nm, consistent with the typical particle size distribution of exosomes. The concentration of exosomes was 7.2 × 10^10^ particles/mL (Figure 1B). Western blot analysis verified the presence of the exosome markers CD9 and CD63 and the absence of the endoplasmic reticulum protein calnexin, supporting the purity of the exosome agent (Figure 1C). Total RNA was extracted from the isolated urinary exosomes, yielding an average of approximately 15–45 ng of RNA per mL of urine. qRT‐PCR analysis of miR‐217 yielded Ct values ranging from 24 to 28, which fell within the reliable detection range for exosomal miRNA quantification. Importantly, qRT‐PCR data suggested that miR‐217 expression was remarkably upregulated in urinary exosomes of DKD patients in the DKD group relative to the normal and DM groups. Meanwhile, the expressions of miR‐217 in urinary exosomes were higher in both DM and DKD groups than in normal groups, and the effect was more obvious in the DKD‐derived exosomes (DKD‐Exo) group (Figure 1D). These findings confirmed the successful isolation of DKD‐Exo and suggest that miR‐217 is enriched in these vesicles.

### 3.2 DKD‐exo promote ferroptosis in renal podocytes

**FIGURE 2:**
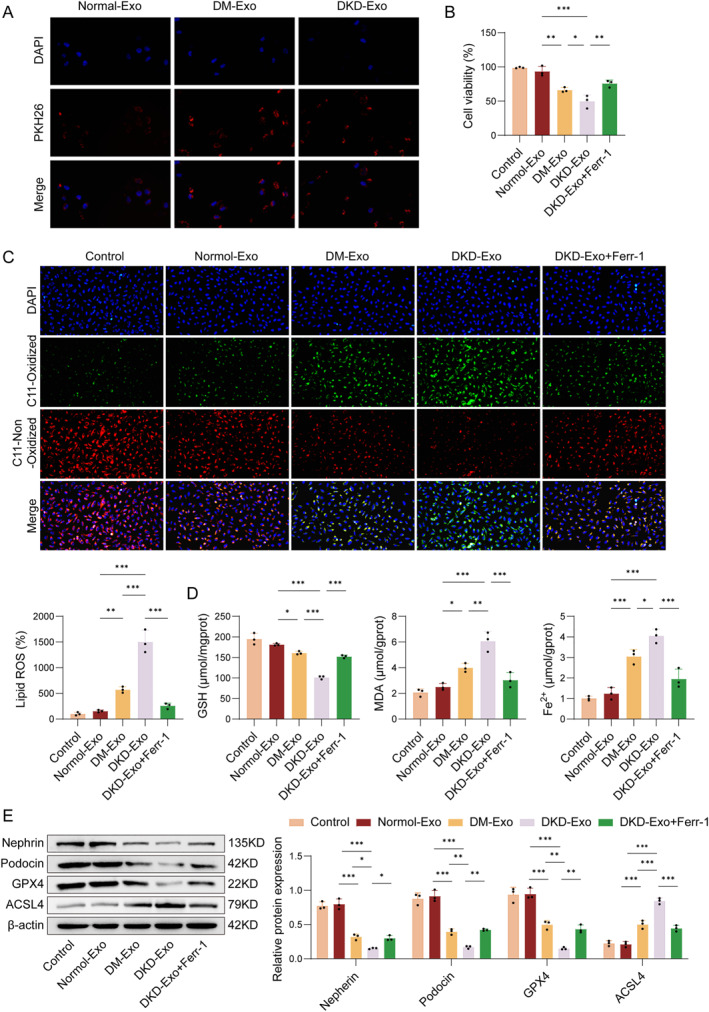
Effects of DKD‐Exo on the viability and ferroptosis of MPC5 cells. (A) Fluorescence microscopy confirmed the uptake of PKH26‐labeled exosomes from the normal‐Exo, DM‐Exo, and DKD‐Exo groups by MPC5 cells. (B) The viability of MPC5 cells exposed to exosomes was assessed using the CCK‐8 assay. (C) The lipid ROS levels were detected by the C11‐BODIPY^581/591^ fluorescent probe. (D) The levels of GSH, MDA, and Fe^2+^ were measured using commercially available assay kits. (E) The expression of nephrin, podocin, GPX4, and ACSL4 was measured by Western blot analysis. *n* = 3, *p < 0.05, **p < 0.01.

Next, we explored the potential of DKD‐derived exosomes (DKD‐Exo) to induce injury in MPC5 podocytes. Cells were treated with DKD‐Exo following pre‐intervention with ferrostatin‐1 (Fer‐1), an inhibitor of ferroptosis. Uptake of exosomes was confirmed by PKH26 staining, with red fluorescently labeled exosomes observed inside MPC5 cells whose nuclei were counterstained with DAPI (blue). No significant difference in internalization was detected among the normal‐Exo, DM‐Exo, and DKD‐Exo groups (Figure 2A). Besides, both DM‐Exo and DKD‐Exo significantly decreased podocyte viability, which was partially rescued by Fer‐1 co‐treatment (Figure 2B). Exposure to DM‐Exo or DKD‐Exo led to reduced glutathione (GSH) levels and elevated lipid ROS, MDA, and Fe^2+^ content in podocytes, and the addition of Fer‐1 intervention could partially reverse the effects of DKD‐Exo (Figure 2C,D). Western blot analysis revealed that DKD‐Exo treatment significantly downregulated the expression of podocyte‐specific proteins (nephrin and podocin) as well as the anti‐ferroptotic protein GPX4, while it obviously upregulated the expression of pro‐ferroptotic marker ACSL4 compared with the normal‐Exo group. These effects were also partially reversed upon Fer‐1 administration (Figure 2E). Collectively, these findings indicated that DKD‐Exo promote ferroptosis in renal podocytes.

### 3.3 DKD urinary exosomes regulate podocyte ferroptosis via miR‐217

**FIGURE 3:**
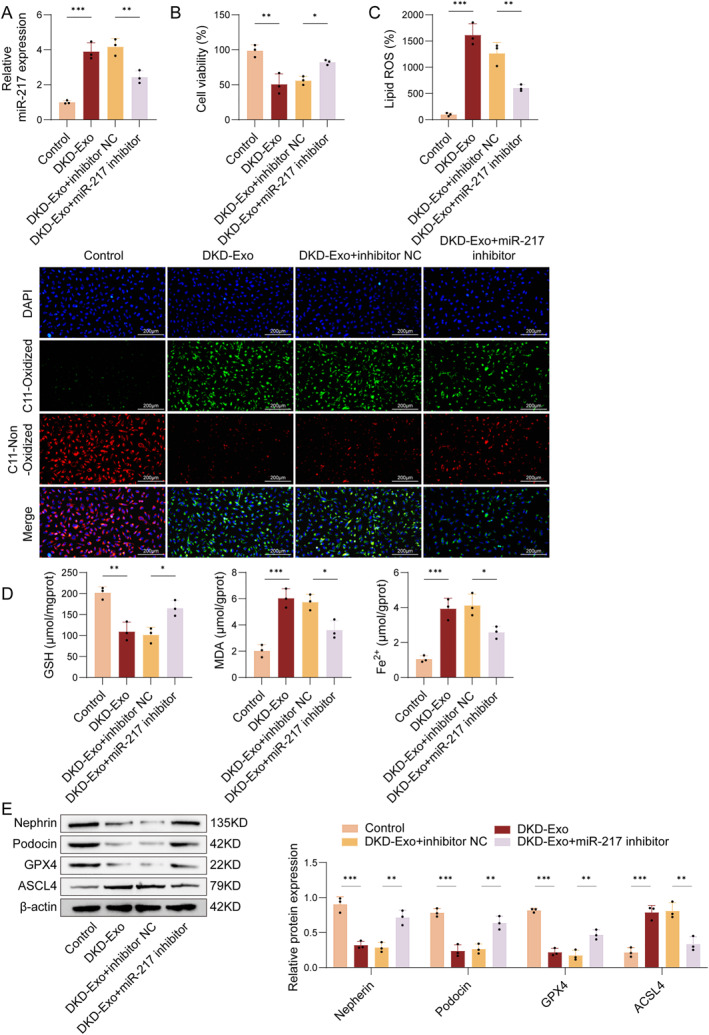
Effects of exosomal miR‐217 on the viability and ferroptosis of MPC5 cells. (A) The mRNA levels of miR‐217 were assessed by qRT‐PCR. (B) The viability of MPC5 cells was assessed using the CCK‐8 assay. (C) The lipid ROS levels were measured using the C11‐BODIPY^581/591^ fluorescent probe. (D) The contents of GSH, MDA, and Fe^2+^ were determined via commercially available assay kits. (E) The expression of nephrin, podocin, GPX4, and ACSL4 was detected through Western blot. *n* = 3, *p < 0.05, **p < 0.01.

To determine whether miR‐217 mediates exosome‐induced ferroptosis, MPC5 cells were cocultured with DKD‐Exo that had been pretreated with 50 nM inhibitor control (NC) or miR‐217 inhibitor. As illustrated in Figure 3A, miR‐217 expression was notably elevated in MPC5 cells following DKD‐Exo treatment compared to the control group. Administration of the miR‐217 inhibitor abolished the DKD‐Exo‐induced upregulation of miR‐217 in MPC5 cells relative to the DKD‐Exo + inhibitor NC group. A marked inhibition in cell viability was found in DKD‐Exo‐treated MPC5 cells, and this reduction was reversed by miR‐217 inhibitor treatment (Figure 3B). In addition, DKD‐Exo significantly decreased GSH content and increased lipid ROS, MDA, and Fe^2+^ content. In contrast, miR‐217 inhibition showed the opposite results (Figure 3C,D). Figure 3E further revealed that DKD‐Exo downregulated the expression of nephrin, podocin, and GPX4, while upregulating ACSL4 expression. These effects were significantly reversed by the miR‐217 inhibition. These observations indicated that exosomal miR‐217 plays a critical role in promoting podocyte ferroptosis induced by DKD‐Exo

### 3.4 DKD‐exo regulates the SIRT1/Nrf2 pathway via targeting miR‐217

**FIGURE 4:**
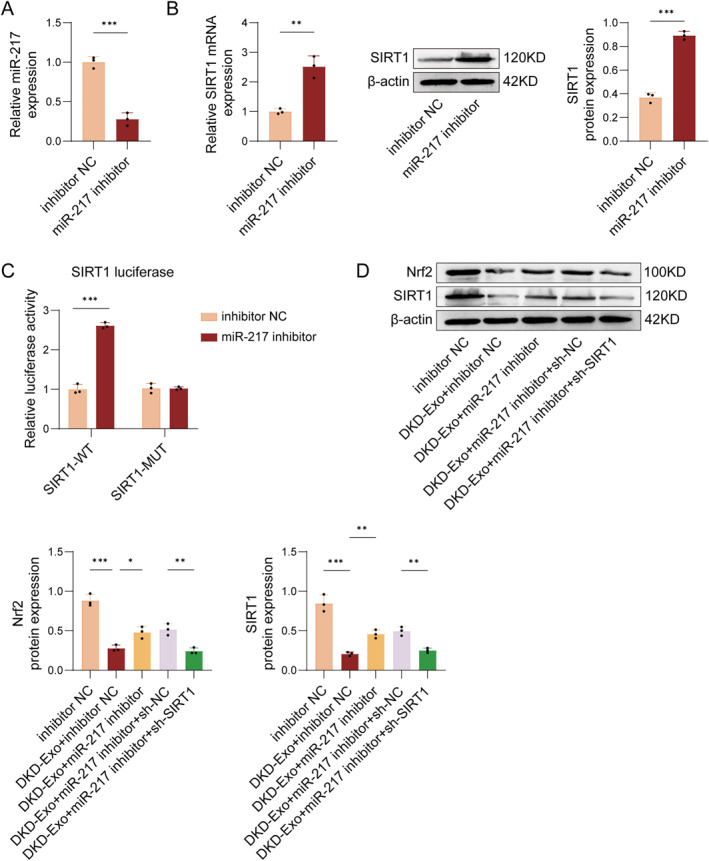
Exosomal miR‐217 regulates the SIRT1/Nrf2 pathway in MPC5 cells exposed to DKD‐Exo. (A) The mRNA level of miR‐217 was determined by qRT‐PCR analysis. (B) The expressions of SIRT1 protein and mRNA levels in MPC5 cells were detected by Western blot and qRT‐PCR. (C) The binding sites between miR‐217 and SIRT1 were predicted by Dual‐Luciferase Reporter Assay. (D) SIRT1 and Nrf2 expressions in MPC5 cells were evaluated via Western blot. *n* = 3, *p < 0.05, **p < 0.01.

To illustrate the molecular mechanism of exosomal miR‐217 in regulating ferroptosis in DKD, MPC5 cells were cocultured with DKD‐Exo pre‐treated with inhibitor control (NC) or miR‐217 inhibitor at 50 nM. qRT‐PCR and/or Western blot assay revealed that miR‐217 inhibition led to decreased miR‐217 expression and increased SIRT1 levels in MPC5 cells (Figure 4A,B). A Dual‐Luciferase Reporter Assay confirmed that the relative luciferase activity of SIRT1‐WT in the miR‐217 inhibitor group was notably higher than that in the inhibitor NC group. In contrast, there was no significant alteration in the relative luciferase activity of SIRT1‐Mut, indicating a targeting interaction between miR‐217 and SIRT1 (Figure 4C). Additionally, Western blot analysis demonstrated that DKD‐Exo downregulated SIRT1 and its downstream effector Nrf2, an effect that was reversed by the inhibition of miR‐217. In contrast, SIRT1 knockdown abolished the upregulating effect of miR‐217 inhibition on SIRT1 expression (Figure 4D). These findings indicated that DKD‐Exo targets the SIRT1/Nrf2 pathway through miR‐217.

### 3.5 DKD‐exo induces MPC5 cells ferroptosis by targeting the miR‐217/SIRT1/Nrf2 axis

**FIGURE 5:**
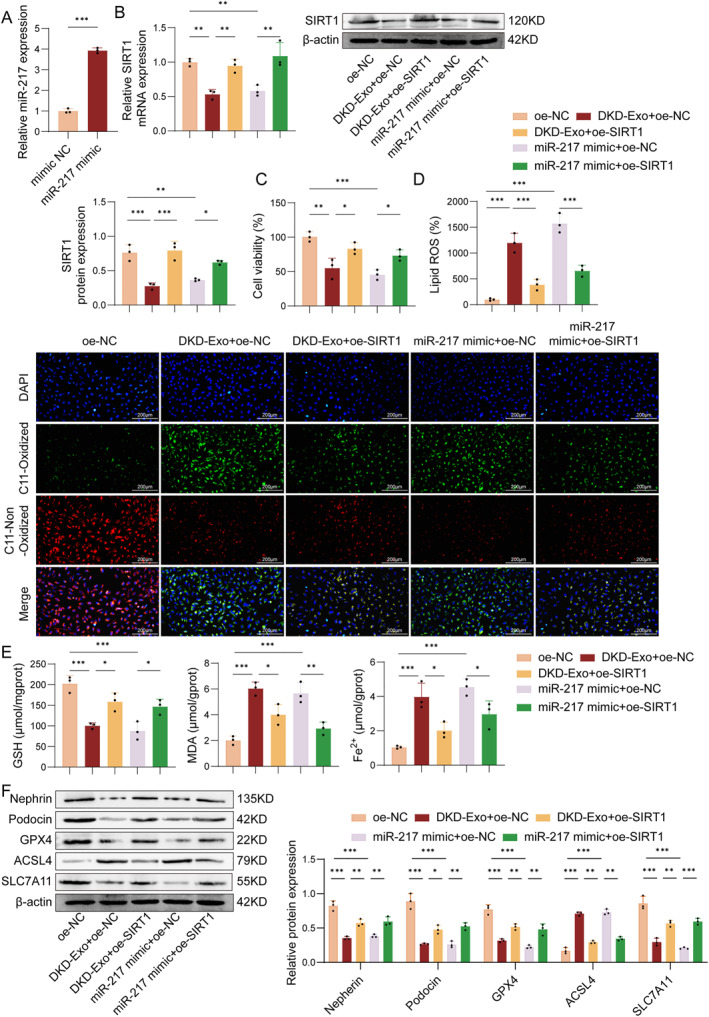
miR‐217/SIRT1/Nrf2 axis mediates DKD‐Exo‐induced podocyte ferroptosis. (A) The relative miR‐217 expression was assessed by qRT‐PCR. (B) The mRNA and protein levels of SIRT1 were detected by qRT‐PCR and Western blot. (C) MPC5 cell viability was assessed via the CCK‐8 assay. (D) The lipid ROS levels were measured using C11‐BODIPY^581/591^ fluorescent probe. (E) The contents of GSH, MDA, and Fe^2+^ were determined via commercially available assay kits. (F) The expression of nephrin, podocin, GPX4, and ACSL4 was measured by Western blot. *n* = 3, *p < 0.05, **p < 0.01.

To investigate the functional role of the miR‐217/SIRT1/Nrf2 axis, we overexpressed miR‐217 and SIRT1 in MPC5 cells. As presented in Figure 5A, overexpression miR‐217 effectively upregulated miR‐217 expression in renal podocytes. Compared with the overexpression negative control (oe‐NC) group, DKD‐Exo treatment decreased SIRT1 expression, which was abolished after SIRT1 overexpression (oe‐SIRT1). Similarly, transfection with miR‐217 mimic reduced SIRT1 expression, and this reduction was rescued by co‐transfection with oe‐SIRT1 (Figure 5B). Furthermore, both DKD‐Exo and miR‐217 mimic exacerbated ferroptosis, manifested by reduced cell viability (Figure 5C), elevated lipid ROS, decreased GSH content, and increased levels of MDA and Fe^2+^. However, these effects were attenuated by SIRT1 overexpression (Figure 5D,E). Results in Figure 5F revealed that the overexpression of SIRT1 reversed the effects of DKD‐Exo and miR‐217 mimic on the expression of nephrin, podocin, SLC7A11, GPX4, and ACSL4, relative to the oe‐NC group (Figure 5F). Collectively, these observations demonstrated that DKD‐Exo promoted ferroptosis in renal podocytes via the miR‐217/SIRT1/Nrf2 axis, underscoring the important role of this pathway in podocyte ferroptosis.

### 3.6 DKD exosomes exacerbate diabetic nephropathy in mice via miR‐217/SIRT1/Nrf2‐dependent ferroptosis

**FIGURE 6:**
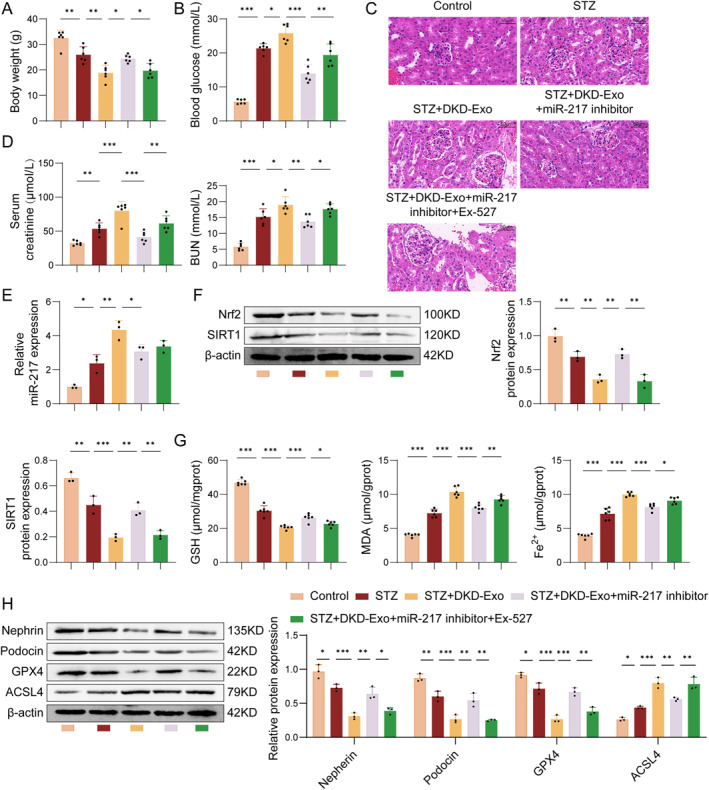
Effects of DKD‐Exo, miR‐217 inhibitor, and the SIRT1 inhibitor on renal injury in the kidney of STZ‐induced mice. The body weight (A) and blood glucose level (B) were assessed. (C) The histopathological changes of renal tissues were evaluated via H&E staining. (D) The Scr and BUN levels were detected via the corresponding detection kits. (E) The miR‐217 mRNA levels of miR‐516b‐5p were determined by qRT‐PCR. (F) The protein levels of SIRT1 and Nrf2 were assessed by Western blot. (G) GSH, MDA, and Fe^2+^ levels were determined by corresponding detection kits. (H) Protein expression of nephrin, podocin, SLC7A11, GPX4, and ACSL4 was detected by Western blot. *n* = 3, *p < 0.05, **p < 0.01.

To explain the function of the miR‐217/SIRT1/Nrf2 pathway in a STZ‐induced DKD mouse model, we evaluated the effects of DKD‐Exo, miR‐217 inhibitor, and the SIRT1 inhibitor Ex‐527 on renal injury. Compared with the control group, mice in the STZ and STZ + DKD‐Exo groups exhibited an obvious reduce in body weight, elevated blood glucose levels, and increased Scr and BUN levels (Figure 6A,B,D). Histological examination revealed pathological alterations in the STZ group, including glomerular hypertrophy, narrowed Bowman's space, nuclear deformation, mesangial matrix expansion, and disorganization of pericapsular cells (Figure 6C). These findings indicated progressive renal structural damage, with mesangial matrix expansion serving as a key feature of fibrotic remodeling. These findings were further exacerbated in the STZ + DKD‐Exo group. Moreover, treatment with the miR‐217 inhibitor ameliorated these alterations, as evidenced by the partial restoration of body weight, reduced blood glucose, enhanced renal function indicators, and alleviated renal histopathological injury in the STZ + DKD‐Exo group. Notably, miR‐217 inhibition reduced mesangial matrix expansion and ameliorated glomerular structural abnormalities, suggesting attenuation of fibrosis‐related pathological changes. However, co‐administration of the SIRT1 inhibitor Ex‐527 partially reversed the protective effects conferred by miR‐217 inhibition. As illustrated in Figure 6E,F, the expression of miR‐217 was enhanced, whereas SIRT1 and Nrf2 levels were reduced in the STZ group compared to the control group, which were further enhanced following DKD‐Exo treatment. Inhibition of miR‐217 partially counteracted the regulatory effects of DKD‐Exo on miR‐217, SIRT1, and Nrf2 expressions, and we observed the opposite results in the Ex‐527 administration group. Furthermore, the STZ group showed decreased GSH levels and increased levels of MDA and Fe^2+^ (Figure 6G). The expression of nephrin, podocin, and GPX4 was downregulated, whereas ACSL4 expression was upregulated in STZ‐induced DKD mice, further supporting the presence of fibrotic pathology (Figure 6H). Notably, these changes were more pronounced after DKD‐Exo treatment. Meanwhile, the effects of DKD‐Exo on nephrin, podocin, GPX4, and ACSL4 expression was partially reversed by miR‐217 inhibition, whereas the protective roles of miR‐217 inhibition was further diminished by Ex‐527 treatment. In conclusion, DKD‐Exo may deliver miR‐217 to inhibit the SIRT1/Nrf2 signaling pathway, thereby promoting oxidative stress and podocyte ferroptosis, which exacerbates the progression of DKD.

## 4 DISCUSSION

Diabetic kidney disease (DKD) is among the most severe microvascular complications linked to diabetes mellitus, characterized by persistent proteinuria and/or progressive decline in renal function.^17^ Given the complexity of its pathogenesis and the current limitations in clinical interventions, there is an immediate necessity to develop new therapeutic strategies. In recent years, exosome‐based therapies have demonstrated considerable potential for DKD therapies. Exosomes contain miRNAs that can be delivered to recipient cells via body fluid circulation and participate in regulating pathophysiological processes.^18^ Notably, urinary exosomal miRNAs facilitate intercellular communication among different segments of the nephron and play a pivotal role in the development of DKD.^19^ Focusing on the urinary exosome miR‐217 in DKD patients, this study systematically elucidated for the first time that miR‐217 accelerates the progression of DKD by promoting ferroptosis in podocytes by targeting the SIRT1/Nrf2 pathway. Our discoveries not only enhance the comprehension of the molecular mechanisms underlying DKD but also provide a novel foundation for exosomal miRNA‐targeted therapy.

Exosomes are extracellular vesicles that facilitate the intercellular transfer of genetic material, lipids, and cell‐specific proteins.^20^ Numerous studies have implicated exosomes and their cargo of signaling molecules in the pathogenesis of various diseases, including tumors,^21^ pulmonary fibrosis,^22^ and immune disorders.^23^ Although the pathogenesis of DKD remains incompletely understood, growing evidence supports the involvement of exosomes in its pathophysiology, highlighting their potential as novel biomarkers for early diagnosis.^24^ Urinary exosomes are primarily derived from renal tissues, including the renal parenchyma and epithelial cells.^25^ Previous studies have investigated the diagnostic relevance of specific miRNAs in urinary exosomes. For instance, Zheng et al. evaluated the expression and clinical diagnostic value of miR‐136‐5p in DKD.^26^ Moreover, Zhao et al. identified miRNA‐4534 as a new diagnostic biomarker for diabetic nephropathy.^27^ In the present study, urine samples were collected from diabetic patients, DKD patients, and healthy volunteers. Exosomes were isolated, characterized, and subsequently transfected into MPC5 cells. Accumulating evidence indicates that miR‐217 plays a significant role in diabetic nephropathy. Ren et al. reported elevated levels of miR‐217 in type 2 diabetes mellitus (T2DM) patients.^28^ Additionally, upregulation of miR‐217 was observed in HG‐stimulated podocytes, further supporting its potential regulatory role in DKD.^10^ Consistent with these findings, our results demonstrated an obvious elevation of miR‐217 in DKD‐Exo, MPC5 cells, and STZ‐induced diabetic mice. Moreover, treatment with exosomes derived from DKD patients (DKD‐Exo) exacerbated renal dysfunction and histopathological damage in a DKD mouse model. Early DKD is often associated with weight loss and hyperglycemia, which were also observed in STZ‐induced mice and DKD‐Exo‐treated mice. Notably, these pathological changes were mitigated by the inhibition of miR‐217, suggesting a promotive role of miR‐217 in DKD progression. Ferroptosis, an emerging form of regulated cell death, has been increasingly implicated in podocyte injury. Research studies by Xiong et al. and Huang et al. demonstrated that therapeutic interventions could attenuate diabetic nephropathy by suppressing ferroptosis, as indicated by reduced levels of ROS, MDA, Fe^2+^, and ACSL4, along with increased GSH.^29^, ^30^ In alignment with these reports, our study showed that DKD‐Exo treatment significantly decreased GSH, upregulated lipid ROS, MDA, and Fe^2+^, and reduced the expression of nephrin, podocin, and GPX4 in podocytes. These effects were partially reversed by either miR‐217 inhibition or Fer‐1 treatment, indicating that DKD‐Exo mediate podocyte injury via miR‐217‐dependent ferroptosis regulation. These observations offer new insights into the role of exosomal miRNAs in DKD and demonstrate possible therapeutic targets.

A growing body of evidence highlights the binding sites between miR‐217 and the 3′‐UTR of SIRT1.^31^ miR‐217 suppresses osteosarcoma proliferation, migration, and invasion via directly targeting SIRT1, further confirming its role as a negative regulator of SIRT1.^32^ As a key member of the sirtuin family, SIRT1 modulates critical processes such as inflammation, autophagy, and ferroptosis.^33^, ^34^ Notably, SIRT1 is closely linked to the activation of Nrf2. Prior studies showed that overexpression of SIRT1 or Nrf2 mitigates erastin‐induced mitochondrial damage and ferroptosis in HT22 cells.^35^ Additionally, fucoxanthin attenuates oxidative stress, renal dysfunction, and fibrosis in diabetic kidneys by activating the SIRT1/Nrf2 pathway.^36^ According to these findings, we hypothesized that DKD‐Exo mediate miR‐217 via the SIRT1/Nrf2 axis. Our experiments revealed that DKD‐Exo significantly decreased SIRT1 and Nrf2 protein levels, which was reversed by the miR‐217 inhibitor. Conversely, SIRT1 knockdown abolished the miR‐217 inhibitor‐mediated upregulation of SIRT1 and Nrf2. Similarly, SIRT1 overexpression counteracted the suppression of w by miR‐217 mimic. Strikingly, SIRT1 inhibitors abolished the protective effects of the miR‐217 inhibitor against podocyte ferroptosis, cellular injury, and renal histopathological damage. This study demonstrates that miR‐217 in DKD exosomes directly inhibits SIRT1, downregulating the Nrf2 pathway and ultimately promoting podocyte ferroptosis and renal dysfunction. Our findings not only underscore the pivotal role of the miR‐217/SIRT1/Nrf2 axis in DKD but also align with existing evidence supporting the protective role of SIRT1/Nrf2 signaling in diabetic complications.

In summary, this study elucidates a novel mechanism by which miR‐217 derived from urinary exosome of DKD patients promotes podocyte ferroptosis through suppression of the SIRT1/Nrf2 axis. These findings advance our understanding of DKD pathogenesis and highlight the broader role of exosomal miRNAs in disease progression. However, this study also has several limitations. Firstly, podocyte injury may be associated with numerous genes or interconnected molecular pathways, instead of being regulated by a single gene. Secondly, although the STZ‐induced DKD model is widely used, it does not fully recapitulate the metabolic and inflammatory characteristics of human DKD, particularly the progressive nature and complex pathophysiology. Therefore, our findings should be further validated in more clinically relevant models.

## AUTHOR CONTRIBUTIONS

Xiu Li guaranteed the integrity of the entire study. Xin Du, Hao Deng, and Xiu Li designed the study and literature research. Haiying Zhang, Xinyi Huang, and Xiu Li defined the intellectual content. Hongbin Guo, Haiying Zhang, Shaoqing Wang, and Xiu Li performed experiment. Xin Du, Yubo Jiang, Hongbin Guo, Hao Deng, and Xinyi Huang collected the data. Yubo Jiang, Hongbin Guo, Hao Deng, Xinyi Huang, and Xiu Li analyzed the data. Xin Du, Yubo Jiang, Hongbin Guo, and Xiu Li wrote the main manuscript and prepared figures. All authors reviewed the manuscript.

## CONFLICT OF INTEREST STATEMENT

The authors declare that they have no known competing financial interests or personal relationships that could have appeared to influence the work reported in this paper.

## ETHICS STATEMENT

All animal procedures and protocols were followed using the guidelines set by the Institutional Animal Care and Use Committee of the 3RD Affiliated Hospital of Chengdu Medical College Chengdu Pidu District People's Hospital (2024‐No.33).

## Data Availability

The datasets used or analyzed during the current study are available from the corresponding author on reasonable request.
